# Kyrieleis like plaques - atypical presentation of ocular Behcet’s disease

**DOI:** 10.22336/rjo.2021.75

**Published:** 2021

**Authors:** Ashok Kumar, Akanksha Sahu, Jaya Kaushik, Rohit Bhanot, Amit Arora

**Affiliations:** * Armed Forces Medical College, Pune, India; ** Department of Ophthalmology, Military Hospital, Kirkee, Pune, India

**Keywords:** Behcet’s disease, Kyrieleis-like plaques, HLA-B51, ocular

## Abstract

**Purpose:** To report an unusual presentation with kyrieleis like plaques in a patient with ocular Behcet’s disease.

**Case presentation:** A 28-year-old young male presented with blurring of vision in the left eye, fundus examination revealing focal segmental intra-arterial plaques involving all branches of retinal artery characteristics of kyrieleis-like plaques with no features of retinitis in retinal periphery and mild vitritis. All routine investigations were normal, the patient testing positive for HLA-B51 marker, diagnosed as a case of ocular Bechet’s disease and managed with oral steroids and immunosuppressive agents.

**Results:** The patient had good recovery of vision with substantial resolution of intra-arterial plaques.

**Conclusion:** Ocular Bechet’s disease can present with uncommon features of kyrieleis like plaques. Ophthalmologists need to be vigilant for the presence of such potential ocular manifestations as the likely initial presentation in order to obtain early diagnosis and initiate timely management.

## Introduction

Kyrieleis plaques were originally described in association with ocular tuberculosis and are most commonly seen in patients of toxoplasmic retinochoroiditis. The similar plaques have also been reported with Rickettsia conorii, Mycobacterium tuberculosis, Syphilis, and Herpes Simplex infections [**1**]. The exact pathogenesis of Kyrieleis plaques is still not known, but it has been suggested that they may represent an immunological response to an infectious agent resulting in the deposition of immune cells within or adjacent to the vessel wall [**2**]. 

We reported the presence of Kyrieleis-like plaques, an atypical initial presentation of ocular Bechet’s disease in a young male. To the best of our knowledge, this rare association has not been previously reported.

## Case report

**Fig. 1:**
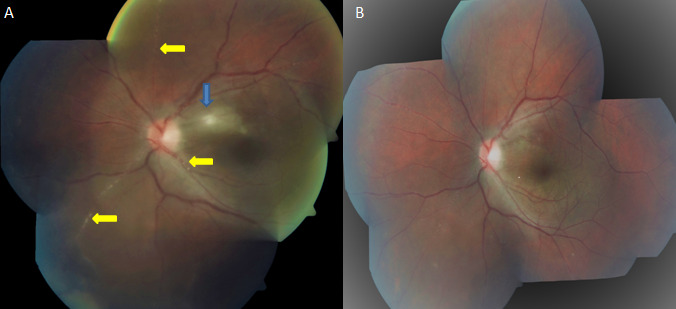
**A** Fundus picture of the left eye showing mild vitreous haze, disc hyperaemia, half disc diameter greyish white lesion in macular area suggestive of retinitis (blue arrow) and focal segmental intra-arterial plaques involving all branches of retinal artery (yellow arrows) characteristic of kyrieleis-like plaques **1B.** Fundus picture at 4 weeks post initiation of treatment revealing resolution of retinitis patch and significant decrease in kyrieleis-like plaques

A 28-year-old male patient, with no known systemic co-morbidity, presented to the ophthalmology outpatient department with mild diminution of vision in the left eye for a period of 10 days. On evaluation, he had a best corrected visual acuity of 6/6 in the right eye, 6/18 in the left eye, and quiet anterior segment in both eyes. On fundus evaluation, the left eye revealed mild vitreous haze, disc hyperaemia, half disc diameter greyish white lesion in macular area suggestive of retinitis (blue arrow, **Fig. 1A**) and focal segmental intra-arterial plaques involving all branches of retinal artery (yellow arrows, **Fig. 1A**), characteristic of kyrieleis-like plaques, with no features of retinitis in retinal periphery. The ocular examination of the right eye was completely unremarkable. Fundus fluorescein angiography revealed fern-like hyperfluorescence involving small and medium retinal vessels typical of Bechet’s disease. In view of the presence of kyrieleis like plaques, he was investigated to rule out systemic infection in the form of toxoplasma, tuberculosis, cytomegalovirus retinitis, herpes simplex, syphilis, and rickettsia infection. The detailed work-up revealed normal IgM as well as IgG titres for toxoplasma, all other investigations being also normal. On further investigations, HLA-B51 genetic marker was positive for the patient. He was evaluated by an immunologist, but no systemic features of Bechet’s disease were elicited and diagnosed as a case of isolated ocular Bechet’s disease. He was started on high dose oral steroids (prednisone 2 mg/kg), initially with addition of azathioprine, with significant improvement in visual acuity to 6/6P, resolution of retinitis lesion and significant decrease in kyrieleis like plaques (**Fig. 1B**) at four weeks post initiation of treatment.

## Discussion

Kyrieleis like plaques are focal segmental intra-arterial plaques resembling arterial emboli commonly seen in ocular toxoplasmosis, but can occur in tuberculosis, varicella-zoster infection, cytomegalovirus (CMV) retinitis, ocular syphilis, and rickettsial disease [**1**].

It results mainly from deposition of immune cells within or adjacent to the vessel wall [**3**]. It has also been hypothesized to result from extravasation of blood lipids into the arterial walls following retinal arterial damage. Wise et al. suggested that these plaques resulted from arteriosclerosis, but few others found them to be a result of the migration of exudates from active choroiditis to periarterial sheaths [**4**]. These intra-arterial plaques may heal on treatment with corticosteroids or antibiotics depending on aetiology [**5**]. 

Behcet’s disease is typically characterized by a triad of oral ulcers, genital ulcers, and ocular lesions [**6**]. It typically presents as bilateral panuveitis with predominant posterior segment manifestations in the form of vitritis, retinitis, vasculitis and papillitis with potentially blinding complications [**7**]. Kyrieleis like plaques has not been documented as a feature of ocular Behcet’s disease. In the present case, isolated uniocular presentation with kyrieleis like plaques was seen without any significant anterior segment inflammation, which responded considerably to high dose oral steroids and immunomodulator drugs. 

## Conclusion

Our case emphasized that Bechet’s disease can present with atypical ocular features like intraarterial plaques. Therefore, the presence of kyrieleis like plaques in an individual with signs of uveitis, should prompt an ophthalmologist for early systemic investigation, to diagnose and treat underlying aetiology with good visual recovery. 

**Conflict of Interest statement**

The author(s) declares that they have no potential conflicts of interest.

**Informed Consent and Human and Animal Rights statement**

Informed consent has been obtained from all individuals included in this study.

**Authorization for the use of human subjects**

Ethical approval: The research related to human use complies with all the relevant national regulations, institutional policies, is in accordance with the tenets of the Helsinki Declaration, and has been approved by the review board of Military Hospital, Kirkee, Pune, India.

**Acknowledgements**

Nil.

**Sources of Funding**

None to declare.

**Disclosures**

None.
